# A Recombinant Horseshoe Crab Plasma Lectin Recognizes Specific Pathogen-Associated Molecular Patterns of Bacteria through Rhamnose

**DOI:** 10.1371/journal.pone.0115296

**Published:** 2014-12-26

**Authors:** Sim-Kun Ng, Yu-Tsyr Huang, Yuan-Chuan Lee, Ee-Ling Low, Cheng-Hsun Chiu, Shiu-Ling Chen, Liang-Chi Mao, Margaret Dah-Tsyr Chang

**Affiliations:** 1 Institute of Molecular and Cellular Biology & Department of Medical Science, National Tsing Hua University, Hsinchu, Taiwan, Republic of China; 2 Department of Biology, Johns Hopkins University, Baltimore, Maryland, United States of America; 3 Department of Pediatrics, Chang Gung Memorial Hospital, Taoyuan Hsien, Taiwan, Republic of China; 4 Simpson Biotech Co., Ltd., Kuei Shan, Taoyuan County, Taiwan, Republic of China; The Hospital for Sick Children and The University of Toronto, Canada

## Abstract

Horseshoe crab is an ancient marine arthropod that, in the absence of a vertebrate-like immune system, relies solely on innate immune responses by defense molecules found in hemolymph plasma and granular hemocytes for host defense. A plasma lectin isolated from the hemolymph of Taiwanese *Tachypleus tridentatus* recognizes bacteria and lipopolysaccharides (LPSs), yet its structure and mechanism of action remain unclear, largely because of limited availability of horseshoe crabs and the lack of a heterogeneous expression system. In this study, we have successfully expressed and purified a soluble and functional recombinant horseshoe crab plasma lectin (rHPL) in an *Escherichia coli* system. Interestingly, rHPL bound not only to bacteria and LPSs like the native HPL but also to selective medically important pathogens isolated from clinical specimens, such as Gram-negative *Pseudomonas aeruginosa* and *Klebsiella pneumoniae* and Gram-positive *Streptococcus pneumoniae* serotypes. The binding was demonstrated to occur through a specific molecular interaction with rhamnose in pathogen-associated molecular patterns (PAMPs) on the bacterial surface. Additionally, rHPL inhibited the growth of *P. aeruginosa* PAO1 in a concentration-dependent manner. The results suggest that a specific protein-glycan interaction between rHPL and rhamnosyl residue may further facilitate development of novel diagnostic and therapeutic strategies for microbial pathogens.

## Introduction

Lectins are a group of carbohydrate-binding proteins that recognize specific carbohydrate structures and are widely distributed in living organisms. Based on the structural and sequence similarities of the carbohydrate-recognition domains (CRDs) and the ligand-binding specificities [1], animal lectins are classified into various families such as M-type lectins, P-type lectins, C-type lectins, I-type lectins, and S-type lectins (galectins), as well as calnexin, pentraxins, and tachylectins [2]. They play diverse roles in physiological processes, functioning as cell surface receptors [3], mediating interactions between cells during development and differentiation [4], [5], and recognizing foreign molecules during immune responses [6].

The horseshoe crab, an ancient marine arthropod, has survived for more than 500 million years [7]. Its defense system is solely dependent on an innate immune system that requires hemocytes and hemolymph plasma to protect it from pathogens [8]. Horseshoe crab hemolymph plasma contains many soluble defense molecules, such as lectins, C-reactive proteins, and α_2_-macroglobulin [9]. In the Japanese horseshoe crab, there are six types of lectins, Tachylectin-1 (TL-1) to -4 from hemocytes and TL-5A and -5B from plasma. The characteristics of bacterial cell walls required for their recognition have been studied for the past two decades [10]. In the Taiwanese horseshoe crab, two types of lectins, *Tachypleus* plasma lectin 1 (TPL1) and *Tachypleus* plasma lectin 2 (TPL2), have been isolated and characterized as novel hemolymph proteins secreted into the plasma of *T. tridentatus* species [11]. Among the horseshoe lectins, TPL2 shows an 80% sequence identity with TL-3 [12], and both TPL2 and TL-3 show ligand specificity toward lipopolysaccharides (LPSs), particularly *O*-antigen [10], [12].

Native TPL2 (nTPL2) binds three species of bacteria, *Streptococcus pneumoniae* R36A (Gram-positive), *Vibrio parahaemolyticus* (Gram-negative), and *Escherichia coli* Bos-12 (Gram-negative) in a dose-dependent and saturable manner [13]. nTPL2 has seven cysteins in its 128 amino acids, including a free Cys4 that can form intermolecular disulfide bonds, which are essential for LPS-binding activity [10], [13]. nTPL2 consists of differentially glycosylated and partially protease-cleaved forms, which has caused difficulties in determining the exact moiety responsible for bacterial-binding activity [10]. Results from a recombinant TPL2 with a glycosylation site mutation indicate that glycosylation of TPL2 is apparently not important for LPS binding [10].

In this study, we have engineered a recombinant TPL2 with a *C*-terminal His-tag, recombinant horseshoe crab plasma lectin (rHPL), and successfully expressed it in *E. coli*. We found that rHPL possessed novel pathogen and glycan recognition abilities. A specific ligand, L-rhamnose (L-Rha), a 6-deoxy sugar found widely in bacteria and plants, was identified. Rhamnose is a common component of the cell wall and capsule of many pathogenic bacteria, including *Salmonella enterica* serovar Typhimurium [14], *Pseudomonas aeruginosa* [15], and *Mycobacterium tuberculosis* [16]. L-Rha specificity has not been previously reported in the Japanese horseshoe crab lectins.

## Materials and Methods

### Bacterial strains, growth media, and chemical reagents

*Escherichia coli* Top10F′ (Invitrogen) was used for vector construction and DNA manipulation. *E. coli* expression strain Rosetta (DE3) (Novagen) and vector pET23a (Novagen) were used for protein expression. The plasmid pPICZαA-*tpl2* was provided by Dr. Po-Huang Liang (Institute of Biological Chemistry, Academia Sinica, Taipei, Taiwan). *Enterobacteria aerogenes* ATCC 13048, *Listeria monocytogenes* ATCC 7644, *Shigella flexneri* group B ATCC 12022, *Proteus mirabilis* ATCC 7002, *Serratia marcescens* ATCC 8100, and *Staphylococcus aureus* ATCC 33591 were purchased from Creative Microbiologicals, Ltd., Taiwan. *Pseudomonas aeruginosa* PAO1 and *Klebsiella pneumoniae* CG43 were kindly provided by Dr. Hwan-You Chang (Institute of Molecular Medicine, National Tsing Hua University, Hsinchu, Taiwan). Lipopolysaccharides (LPSs) of *E. coli* O26:B6, *E. coli* O55:B5, *P. aeruginosa* sero 10, *Salmonella enterica* serovar typhimurium and L-Rhamnose (L-Rha) monosaccharide were purchased from Sigma. L-Rhamnose-BSA (Rha-BSA) and blood group A-pentasaccharide were purchased from Dextra Laboratories. Ni-Sepharose 6 Fast Flow was purchased from GE Healthcare. All other buffers and reagents were of the highest commercial purity.

### Cloning of rHPLs

A DNA fragment encoding nTPL2 was amplified by PCR using pPICZαA-*tpl2* [10] as the template with primers 5′ *Eco*RI-rHPL (5′ GAATTCGAAGATGACTGCACGTGACAGAC 3′) and 3′ *Not*I-rHPL-6His (5′ GCGGCCGCTTAATGATGATGATGATGATGCTTAATTATTATAATAGGTCC 3′). PCR reactions were carried out with the following PCR program: Stage 1: 95°C for 5 min, 1 cycle; Stage 2: 95°C for 30 sec, 55°C for 30 sec, 72°C for 1 min, 30 cycles; and Stage 3: 72°C for 5 min, 1 cycle. Purified PCR products were digested with *Eco*RI and *Not*Ι and were ligated into the pET23a vector that had been digested with the same restriction enzymes. The recombinant plasmid was transformed into *E. coli* TOP10F′ and confirmed by sequencing.

### Protein expression and purification

The recombinant plasmids were transformed into *E. coli* expression strain Rosetta (DE3) for overexpression. After induction with a final concentration of 0.1 mM isopropyl β-D-1-thiogalactopyranoside (IPTG) at 16°C for 16 h, cells were harvested by centrifugation, and residues were suspended in equilibrium buffer (20 mM Tris-HCl, 200 mM NaCl, and 5 mM imidazole, pH 7.4) supplemented with protease inhibitor (1 mM phenylmethylsulfonyl fluoride) and disrupted by three passages through EmulsFlex-C3 high pressure homogenizer (Avestin) at 15,000 psi. The recombinant proteins were purified using a Ni-Sepharose column according to the manufacturer's instructions. Purified proteins were then concentrated and buffer-exchanged to Tris buffer (20 mM Tris-HCl and 200 mM NaCl, pH 7.4) using an Amicon Ultra-15 centrifugal filter unit (Millipore).

### Far-UV Circular Dichroism

Far-UV CD spectrum of rHPL (25 µM in 5 mM Tris-HCl, pH 7.4) was recorded using an Aviv CD spectrometer (Model 62A, Aviv Biomedical) with a quartz cuvette of 0.1 cm path length. CD spectrum was collected in Far-UV range from 260 nm to 190 nm at 25°C. Each spectrum was the average of 3 scans. The results were expressed as mean residual ellipticity (MRE, [θ]) in deg.cm^2^. dmol^-1^ which was defined as: millidegrees/(path length in mm times the concentration of protein times the number of residues).

### LPS- and bacteria-binding enzyme-linked immunosorbent assays (ELISAs)

A suspension of 50 µl LPS (0.5 µg/well) or bacteria (5×10^7^ cells/well) in coating buffer (0.1 M sodium carbonate-bicarbonate buffer, pH 9.6, for LPS and a 1∶9 [v/v] mixture of chloroform and ethanol for bacteria) was added to the wells of 96-well microplates and incubated at 4°C overnight. The concentrations of bacteria in the cultures were determined by measuring the OD_600_. The number of cells per milliliter was estimated by assuming that 0.1 absorbance was roughly equivalent to 10^8^ cells/ml [13]. After blocking with 3% bovine serum albumin (BSA) in PBS containing 0.05% Tween-20 (PBST) at 37°C for 2 h, the plates were washed with PBST three times. To the washed wells, 50 µl of 1 µM purified rHPL was added and incubated at 37°C for 1.5 h. Tris buffer (20 mM Tris-HCl and 200 mM NaCl, pH 7.4) was added in parallel as a negative control. After washing three times with PBST, the microplates were incubated with monoclonal anti-His (1∶5000; Clontech) in PBST at 37°C for 1 h. Subsequently, horseradish peroxidase–conjugated anti–mouse IgG (1∶5000; Jackson Lab) in PBST was added to the microplates, and, after washing three times with PBST, the plates were incubated at 37°C for 1 h, at which point, 100 µl of 3,3′,5,5′-tetramethylbenzidine substrate was added to each well, washed three times with PBST, and incubated at 37°C for exactly 15 min. Finally, the reaction was terminated by the addition of 100 µl of 2 N H_2_SO_4_. The OD_450_ was read using a spectrophotometer (Bio-Rad iMark Microplate Absorbance Reader). In the inhibition ELISA, 1 µM of rHPL was first incubated with indicated concentration of each inhibitor in a total volume of 50 µl at 37°C for 30 min, and then added into the wells coated with LPS or bacteria and incubated at 37°C for additional 1 h. Subsequently, the unbound protein was washed off with PBST before detection as described above. All ELISA experiments were individually performed at least three times. The values are indicated as the mean ± SD.

### Magnetic Reduction (MR) assay

Because the binding affinity between glycan-binding proteins and ligands is typically low, we used an ultrasensitive MR assay [17], [18] to measure the association between bio-activated (rHPL-conjugated) magnetic nanoparticles (MNPs) and the target bio-molecule (glycan or LPS). Under multiple external AC magnetic fields, MNPs oscillate physically through magnetic interactions with the applied fields and, thus, exhibit an AC magnetic moment that is known as the AC magnetic susceptibility (χ_ac_). When target bio-molecules are added to the bio-activated MNPs, interactions between the target bio-molecule and MNPs causes portions of the MNPs to aggregate and become less able to rotate compared with non-associated MNPs. Consequently, the measured χ_ac_ is reduced. rHPL was conjugated to dextran-coated Fe_3_O_4_ MNP using a bio-functionalization kit (MagQu) [19], [20]. Analytes including L-Rhamnose, D-Galactose (negative control), or LPS of *P. aureginosa* (positive control) were dissolved in 20 mM Tris-HCl, 200 mM NaCl, and 1 mM EDTA (pH 7.4) to a final concentration of 0.01, 0.05, 0.1, 1, 10, and 1000 ng/ml. Then, 80 µl of rHPL-coated MNPs was mixed with 40 µl of diluted analyte solution and vortexed for 15 s. Finally, the mixture was placed in a superconducting quantum interference device (SQUID)-based magnetosusceptometer, XacPro-S104 (MagQu), to measure the real-time χ_ac_ of the mixture at 25°C. The association between rHPL-conjugated MNPs and analytes were determined by quantifying the reduction in χ_ac_, which was defined as MR (%)  =  (χ_ac,o_ – χ_ac,φ_)/χ_ac,φ_ ×100%, where χ_ac,o_ is the signal of MNPs in the absence of analytes and χ_ac,φ_ is the signal after MNPs associated with analytes.

### Antibacterial activity assay

*Pseudomonas aeruginosa* PAO1 and *Staphylococcus aureus* were incubated in Luria-Bertani (LB) broth at 37°C overnight and then subcultured into fresh broth and grown for 4–6 h until log phase. The cultures were collected by centrifugation and then washed three times with 10 mM sodium phosphate buffer (pH 7.4). After washing, the cell counts were determined at OD_600_. A 25-µl sample of 1×10^6^ cells/ml bacteria was mixed with 25 µl buffer or rHPL to generate a final rHPL concentration of 0 µM, 0.47 µM, 0.94 µM, 1.88 µM, 3.75 µM, 7.5 µM, and 15 µM and incubated at 37°C for 4 h. Afterward, one-quarter of the mixtture was applied to LB agar plates and incubated at 37°C for ∼16 h. By counting the number of CFUs and comparing the results with the control plate (100%), cell mortality was calculated.

### Statistical analyses

All statistical analyses were carried out using GraphPad Prism version 5.01 for Windows (GraphPad Software). All results were considered significant at a *P*-value of <0.05.

## Results

### Expression and purification of rHPL

**Figure 1:**
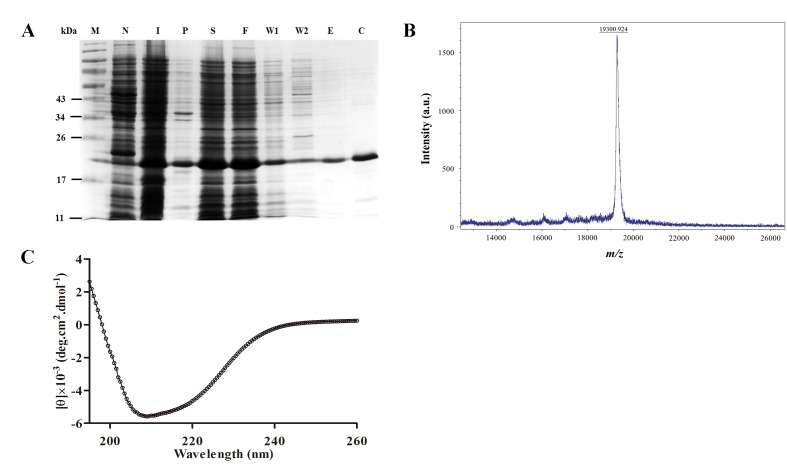
Purification and characterization of rHPL expressed in *E. coli*. (A) After induction with 0.1 mM isopropyl β-D-1-thiogalactopyranoside at 16°C for 16 h, the supernatant containing rHPL was collected by centrifugation and subjected to purification by nickel-column chromatography. Aliquots of each fraction were analyzed by 15% (w/v) SDS-PAGE. The expected molecular weight of rHPL was 19.4 kDa. Lane M: molecular weight marker; Lane N: non-induction; Lane I: induction; Lane P: insoluble pellet; Lane S: supernatant; Lane F: binding flow-through; Lane W1: washing fraction 1; Lane W2: washing fraction 2; Lane E: eluent; Lane C: concentrated fraction. (B) Mass determination of rHPL was performed by MALDI-TOF MS in the electrospray ionization mode. rHPL (100 pmol) was acidified with 0.1% (v/v) formic acid in 50% acetonitrile, and the data were acquired over the mass-to-charge ratio (*m*/*z*) range of 0–26,000 under normal scan resolution (*x* axis), the relative intensity (a.u., arbitrary units) are shown on the *y* axis. The data from each spectra were summed and deconvoluted. (C) Secondary structure of rHPL was measured by Far-UV CD spectrum (260 nm–190 nm) with protein concentration of 25 µM at 16°C.

rHPL was successfully expressed upon induction with 0.1 mM IPTG in *E. coli*, and >50% of the overexpressed rHPL was soluble (Fig. 1A, lanes I and S). rHPL, purified by a nickel-affinity column (Fig. 1A, lane E), was obtained at ∼8 mg/l of culture medium, resulting in a recovery rate of 80.6% with a purity of 93%. In comparison, recombinant TPL2 was reported to be expressed in *P. pastoris* KM71 and purified *via* LPS-Sepharose CL-4B column chromatography with a yield of 1.2 mg/l culture medium [10], which is approximately seven-fold lower than that of rHPL. The molecular weight of rHPL was determined to be 19,301 Da by matrix-assisted laser desorption ionization time-of-flight mass spectrometry (MALDI-TOF MS) (Fig. 1B), which was consistent with the molecular weight estimated from the sequence (19.4 kDa). The purified rHPL was desalted and concentrated using an Amicon protein concentrator (10-kDa cut-off) and subjected to further studies. We successfully produced a recombinant horseshoe crab plasma lectin with improved protein solubility and yield using an *E. coli* expression system.

The secondary structure of rHPL was investigated by Circular Dichroism (CD) as shown in Fig. 1C. A board negative peak was observed from 222 nm to 208 nm, which reflected a mixed secondary structure with higher β–strand content. Since no secondary or tertiary structure of native or recombinant TPL2 is solved yet, rHPL sequence was further input to PredictProtein server (https://www.predictprotein.org/) [21] to predict putative secondary structure features as 14.73% α-helix and 23.26% β–strand in consistent with CD result.

### Binding of rHPL to LPSs

**Figure 2:**
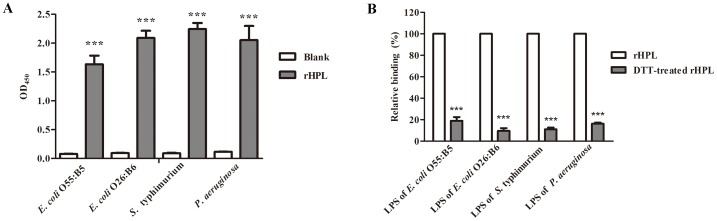
LPS binding activity of rHPL expressed in *E. coli*. A total of 0.5 µg of each LPS was coated on microplate wells and detected with 1 µM rHPL (A) or 1 µM DTT-treated rHPL (B). Monoclonal anti-His (1∶5000) was used to detect the rHPL bound to LPSs. Blank refers to wells containing buffer instead of rHPL. The values are the mean ± SD from triplicate experiments. ***P<0.001 *versus* the corresponding blank data.

To examine LPS-binding activity of rHPL, four different LPSs from *E. coli* O55:B5, *E. coli* O26:B6, *S.* typhimurium, and *P. aeruginosa* sero 10 were immobilized on 96-well microplates. rHPL was added, and binding was measured by ELISA. rHPL significantly bound to all four LPSs (Fig. 2A), quantitative results were shown in S1 Table. In terms of critical role of disulfide bond in rHPL function, LPS/bacteria-binding activities of rHPL in the presence and absence of 5 mM DTT were measured. Fig. 2B showed that binding activities of DTT-treated rHPL to LPSs of *E. coli* O55:B5, *E. coli* O26:B6, *S.* typhimurium, and *P. aeruginosa* sero 10 significantly decreased to respectively 19%, 9.6%, 11.1%, and 16.3% as compared to untreated rHPL. This result strongly indicated that disulfide bond formation facilitated LPS recognition activity of *E. coli* expressed rHPL, similar to the case of yeast-expressed TPL2 [10].

### Binding of rHPL to bacteria

**Figure 3:**
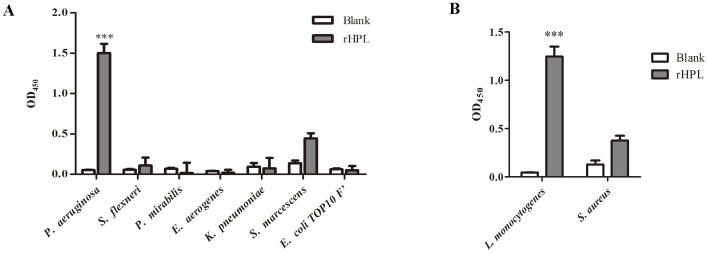
Binding activity of rHPL to laboratory-derived bacteria. (A) Gram-negative and (B) Gram-positive bacterial cells were seeded at 5×10^7^ cells/well, and 1 µM of rHPL was applied to the microplate wells. Subsequently, monoclonal anti-His (1∶5000) was used to detect rHPL bound to bacterial cells. Blank refers to wells containing buffer instead of rHPL. The values are the mean ± SD from triplicate experiments. ***P<0.001 *versus* the corresponding blank data.

**Figure 4:**
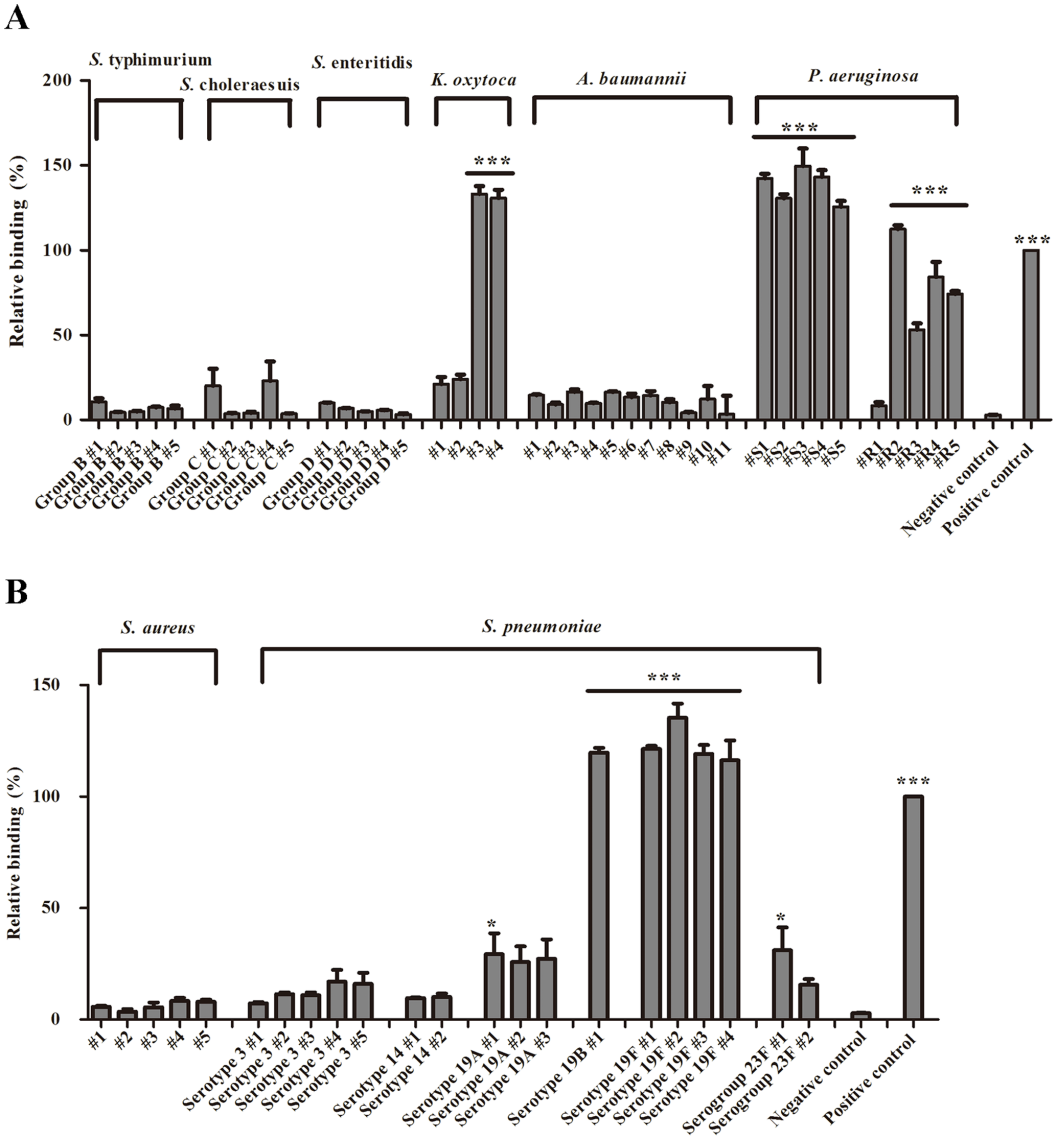
Binding activity of rHPL to clinically-isolated bacteria. (A) Gram-negative and (B) Gram-positive bacterial cells were seeded and rHPL binding was analyzed as in Fig. 3. The values are the mean ± SD from triplicate experiments. Individual sample numbers are indicated. *P. aeruginosa* sero 10 LPS was used as a 100%-binding positive control and *E. coli* Top10F′ was used as negative control. Relative binding percentages are relative to the positive control. *P<0.05 and ***P<0.001 *versus* the corresponding blank data.

To screen for the pathogen recognition patterns of rHPL, seven different laboratory-derived Gram-negative bacteria, *P. aeruginosa* PAO1, *Shigella flexneri*, *Proteus mirabilis*, *Enterobacter aerogenes*, *Klebsiella pneumoniae*, *Serratia marcescens*, and *E. coli* TOP10F′, as well as two Gram-positive bacteria, *Staphylococcus aureus* and *Listeria monocytogenes*, were initially tested using a bacterial-binding ELISA. rHPL significantly bound to *P. aeruginosa*, but not to the other six Gram-negative bacteria (Fig. 3A). Interestingly, rHPL also bound to the Gram-positive bacteria *L. monocytogenes* (Fig. 3B). Quantitative results were shown in S2 Table. In addition, 62 clinically isolated microbial pathogen samples belonging to eight strains, the Gram-negative *Salmonella enterica* serovars typhimurium, choleraesuis, and enteritidis; *Klebsiella oxytoca*; *Acinetobacter baumannii*; and *P. aeruginosa* and the Gram-positive *S. aureus* and *S. pneumoniae*, were screened against rHPL for pathogen-binding activity, with *P. aeruginosa* sero 10 LPS as a 100%-binding positive control. A relative binding percentage of ≥50% was defined as strong binding, and <50% was defined as weak or no binding. Two samples of *K. oxytoca* (#3 and #4) and nine samples of *P. aeruginosa* (#S1, #S2, #S3, #S4, #S5, #R2, #R3, #R4, and #R5), both of which are Gram-negative bacteria, were clearly recognized by rHPL with strong binding activity, whereas no binding of rHPL was detected with the samples from *S.* typhimurium, *S.* choleraesuis, *S.* enteritidis, *A. baumannii*, and *P. aeruginosa* #R1, as well as two samples from *K. oxytoca* (#1 and #2) (Fig. 4A, S3 Table). For Gram-positive bacteria, rHPL strongly bound to all five samples from *S. pneumoniae* serotypes 19B and 19F, and showed weak binding to #1 of both *S. pneumoniae* serotypes 19A and 23F, but no significant binding activity was observed with all samples from *S. aureus* and *S. pneumoniae* serotypes 3 and 14, and two samples from 19A (#2 and #3), and # 2 of 23F (Fig. 4B, S4 Table). It should be noted that rHPL recognition profiles to *K. oxytoca* were significantly different between samples #1, #2 and samples #3, #4. For Gram-negative bacteria, *O*-antigen on LPS is characterized by very high variability in its structure, even within the same species [22]. *O*-antigen of pathogen may contribute to bacterial evasion of host immune responses, which is related to its chain length, and relative amounts of sugar component [23]. Interestingly, antibiogram analysis of *K. oxytoca* showed that samples #1 and #2 were sensitive to antibiotics Ceftriaxone, Ceftazidime, and Cefuroxime, whereas samples #3 and #4 were resistant to these three antibiotics (data not shown), which might be attributed to diffenent *O*-antigen component of these clinically isolated *K. oxytoca* strains. However, exact Rha content in these clinically isolated pathogens still need to be further investigated.

The *O*-antigen in the LPS of certain Gram-negative bacteria has been demonstrated to serve as a specific ligand for TPL2 [10]. However, rHPL was also found to bind to Gram-positive bacteria in this study, indicating that a specific component, possibly a glycan which is present on both lipoteichoic acid (LTA) and LPS of bacterial surfaces, might serve as the rHPL binding ligand.

### Binding of rHPL to Rha

**Figure 5:**
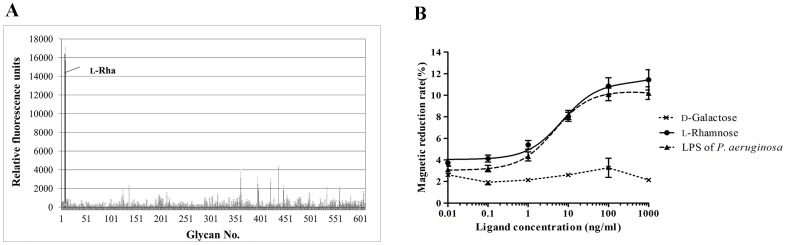
Glycan binding activity of rHPL expressed in E. coli. (A) Glycan microarray analyses were conducted by the Consortium for Functional Glycomics. rHPL at a concentration of 200 µg/ml was used in the analysis, and anti-His (1∶1000) was used as the primary antibody. The values are the mean ± SD from triplicate experiments. (B) Direct binding between L-Rha monohydrate and rHPL was verified by magnetic reduction (MR) assay. rHPL was conjugated on magnetic nanoparticles (MNPs) and the increase of MR signal with the titration of L-Rha concentrations ranging from 0.01 to 1000 ng/ml was measured. LPS of *P. aeruginosa* and D-Galactose were used as positive and negative control respectively.

PAMPs are the major component of the outer membrane of Gram-negative and Gram-positive bacteria. To determine if rHPL bound to PAMPs by recognizing a specific glycan, glycan array screening was carried out by the Consortium for Functional Glycomics using a mammalian printed array (version 5.1; Jul, 2012) [24]. There are 610 synthetic glycans which generally represent the terminal sequences found on *N*-glycans, *O*-glycans, and glycosphingolipids of mammalian tissues. Surprisingly, rHPL bound very specifically and significantly to glycan no. 8, L-Rha monohydrate, on the printed array with an average RFU of 16393 (Fig. 5A). Thus, rHPL might recognize selective bacteria through a specific molecular interaction with the Rha moiety on the bacterial cell surface. Except L-Rha, 8 glycans with average RFU signals higher than 2000 were observed and listed in Table 1. However, glycans no. 360, no. 394, no. 395 and no. 446 had binding variability %CV (100× StDev/average RFU) higher than 20%, hence these binding data might not be reliable [25]. For glycans no. 436, no. 420, no. 136, and no. 559 generally located in *N*-linked glycan terminal structure of mammalian tissues, the average RFU was respectively 4268, 2803, 2141, and 2044, suggesting that they might be further studied for rHPL recognition.

**Table 1:** rHPL binding signals to glycans on CFG array v5.1.

#	Glycan structure	Average RFU	StDev	% CV
8	Rhaα-Sp8	16393	1301	8
436	Galβ1-4GlcNAcβ1-2Manα1-6(GlcNAcβ1-4)(Galβ1-4GlcNAcβ1-4(Galβ1-4GlcNAcβ1-2)Manα1-3)Manβ1-4GlcNAcβ1-4GlcNAc-Sp21	4268	196	5
360	Fucα1-2Galβ1-3GlcNAcβ1-2Manα1-6(Fucα1-2Galβ1-3GlcNAcβ1-2Manα1-3)Manβ1-4GlcNAcβ1-4GlcNAcβ-Sp20	3542	957	27
394	Galα1-3Galβ1-3GlcNAcβ1-2Manα1-6(Galα1-3Galβ1-3GlcNAcβ1-2Manα1-3)Manβ1-4GlcNAcβ1-4GlcNAc-Sp19	2818	756	27
420	Fucα1-2Galβ1-4GlcNAcβ1-2Manα1-6(Fucα1-2Galβ1-4GlcNAcβ1-2Manα1-3)Manβ1-4GlcNAcβ1-4(Fucα1-6)GlcNAcβ-Sp22	2803	444	16
136	Neu5Acα2-6(Galβ1-3)GalNAcα-Sp14	2141	319	15
395	Galα1-3Galβ1-3(Fucα1-4)GlcNAcβ1-2Manα1-6(Galα1-3Galβ1-3(Fucα1-4)GlcNAcβ1-2Manα1-3)Manβ1-4GlcNAcβ1-4GlcNAc-Sp19	2140	427	20
446	Fucα1-2Galβ1-4(Fucα1-3)GlcNAcβ1-2Manα1-6(Fucα1-2Galβ1-4(Fucα1-3)GlcNAcβ1-4(Fucα1-2Galβ1-4(Fucα1-3)GlcNAcβ1-2)Manα1-3)Manβ1-4GlcNAcβ1-4GlcNAcβ-Sp12	2047	433	21
559	Galβ1-4GlcNAcβ1-3Galβ1-4GlcNAcβ1-6(Galβ1-4GlcNAcβ1-3Galβ1-4GlcNAcβ1-2)Manα1-6(Galβ1-4GlcNAcβ1-3Galβ1-4GlcNAcβ1-2Manα1-3)Manα1-4GlcNAcβ1-4GlcNAc-Sp24	2044	325	16

Selective rHPL (200 µg/ml) binding entities including serial number of each glycan (#), glycan structure, binding signals in relative fluorescence units (Average RFU) in decrease order, standard deviation (StDev), and percent coefficient of variance (%CV).

Sp8  = −CH_2_CH_2_CH_2_NH_2_; Sp12  = Asn; Sp 14 =  Thr; Sp19 =  Glu-Asn or Asn-Lys; Sp20 =  Gly-Glu-Asn-Arg; Sp21 = -N(CH_3_)-O-(CH_2_)_2_-NH_2_; Sp22 =  Asn-Ser-Thr; Sp24 =  Lys-Val-Ala-Asn-Lys-Thr.

**Figure 6:**
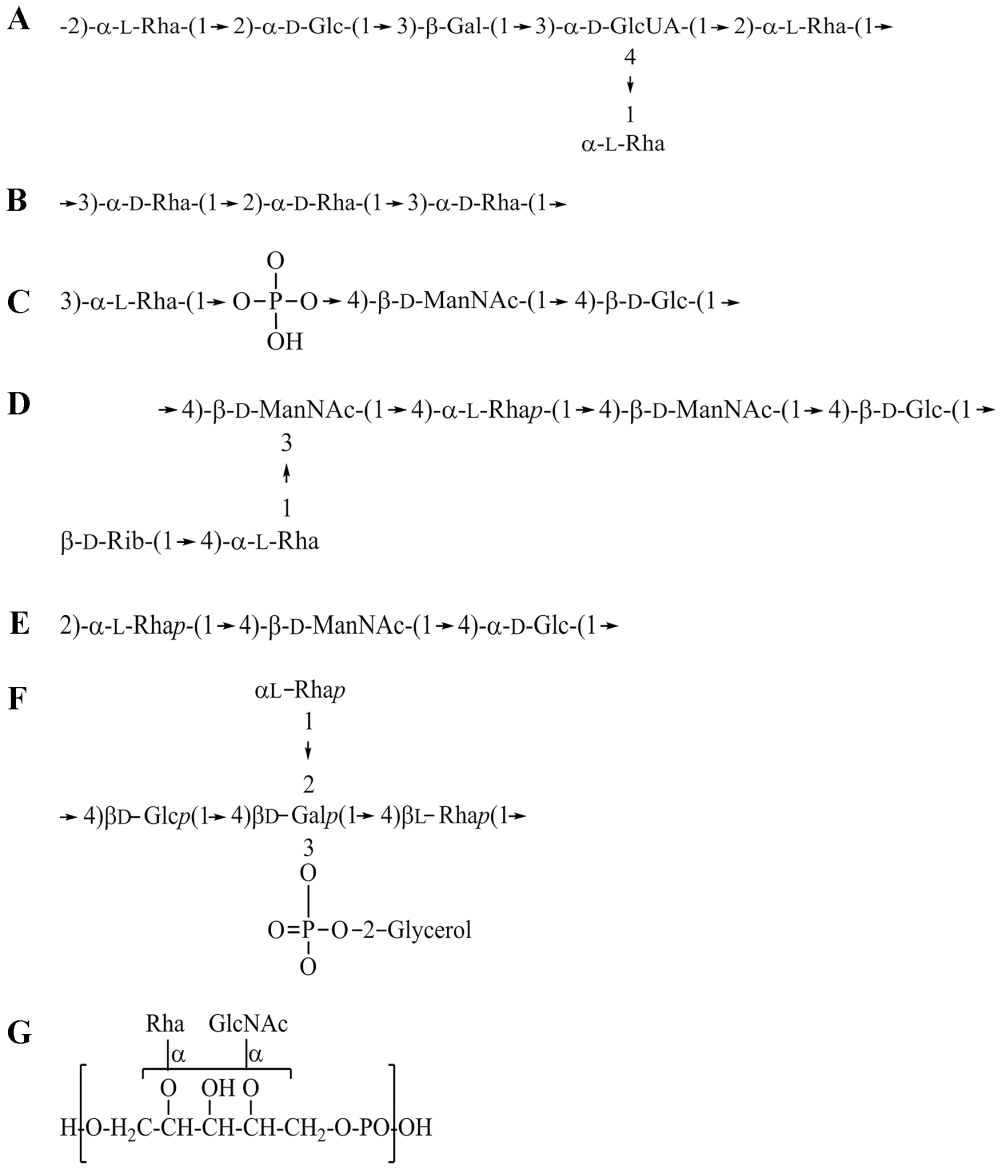
Chemical structures of PAMPs of bacterial pathogens. (A) LPS of *K. oxytoca* strain TMN3. (B) LPS A band of *P. aeruginosa*. (C) Capsule of *S. pneumoniae* serotype 19A. (D) Capsule of *S. pneumoniae* serotype 19B. (E) Capsule of *S. pneumoniae* serotype 19F. (F) Capsule of *S. pneumoniae* serotype 23F. (G) LTA of *L. monocytogenes* ATCC 7644.

Because the binding affinity between glycan-binding proteins and ligands is typically low, with equilibrium dissociation constant values ranging from micromolar to millimolar [26], we used a magnetic reduction (MR) assay [17], [18] to verify direct binding between L-Rha monohydrate and rHPL conjugated on magnetic nanoparticles (MNPs). The MR signal of rHPL-conjugated MNPs showed a sigmoidal increase from 3.8% to 11.3% with the titration of L-Rha concentrations ranging from 0.01 to 1000 ng/ml (Fig. 5B). LPS of *P. aeruginosa* was used as positive control and showed a sigmoidal increase from 3.1% to 10.2%. D-Galactose used a negative control showed no magnetic reduction with the titrating concentrations ranging from 0.01 to 1000 ng/ml. Quantitative results were shown in S5 Table. Thus, this sensitive detection system provided additional evidence for an rHPL-Rha interaction at the molecular level, strongly implicating Rha-containing PAMPs on the bacteria as rHPL recognition sites. Further analyses of reported chemical structures of PAMPs on rHPL-binding bacteria revealed that *K. oxytoca* strain TMN3 [27] and *P. aeruginosa* [15] had three Rha moieties on their LPSs (Fig. 6A and 6B), and *S. pneumoniae* serotype 19A, 19B, 19F and 23F [28], [29] had one or two Rha moiety on their capsules (Fig. 6C to 6F). Interestingly, the LTA of *L. monocytogenes* (ATCC7644) [30] also contained a Rha (Fig. 6G).

### Inhibitory effect of L-Rhamnose and L-Rhamnose-BSA conjugate on rHPL-LPS/bacteria interaction

**Figure 7:**
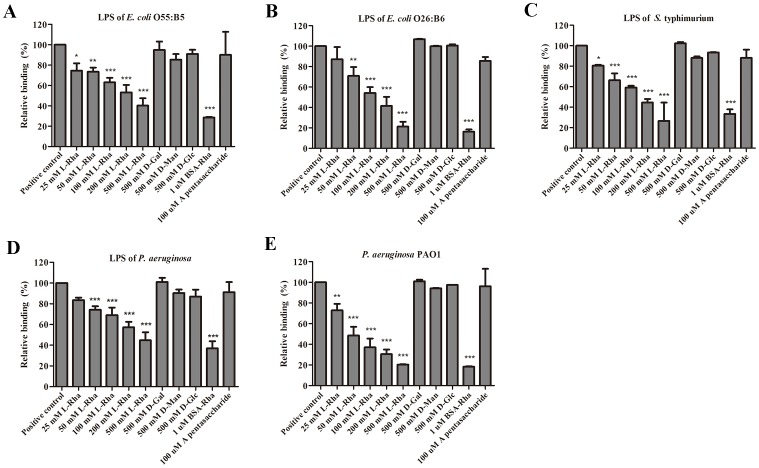
Inhibitory effect of L-Rhamnose monosaccharide and Rha-BSA on rHPL-LPS/bacteria interaction. A total of 0.5 µg of *E. coli* O55:B5 LPS (A), *E. coli* O26:B6 (B), *S.* typhimurium (C), *P. aeruginosa* (D), or 5×10^7^ cells *P. aeruginosa* PAO1 (E) was coated on 96-well microplates and incubated at 37°C for 3 h or at 4°C overnight. The microplates with the immobilized bacteria were washed, and unbound regions were blocked with BSA. Various concentrations of glycans or glycan-protein conjugates were incubated with 1 µM rHPL and then added to microplates. Anti-His (1∶5000) was used to detect rHPL binding to bacterial cells. Blank refers to microplate wells containing only buffer. The values are the mean ± SD from triplicate experiments. *P<0.05, **P<0.01, and ***P<0.001 *versus* the rHPL only group (positive control).

Here L-Rha was used in an inhibitory assay to assess the rHPL-bacteria interaction. To conduct a inhibitory ELISA, 1 µM rHPL was first incubated with 25 mM, 50 mM, 100 mM, 200 mM or 500 mM of L-Rha for 30 min, the mixtures were then added to microplate wells coated with rHPL-recognizing LPSs including *E. coli* O55:B5, *E. coli* O26:B6, *S.* typhimurium, and *P. aeruginosa* sero 10 (Fig. 2), and bacteria *P. aeruginosa* PAO1 (Fig. 3B). As expected, addition of 50 mM, 100 mM, 200 mM or 500 mM of L-Rha could inhibit rHPL to bind to LPSs of *E. coli* O55:B5 (Fig. 7A), *E. coli* O26:B6 (Figure 7B), *S.* typhimurium (Fig. 7C), *P. aeruginosa* sero 10 (Fig. 7D) and bacteria *P. aeruginosa* PAO1 (Fig. 7E) in a concentration-dependent manner. Moreover, addition of 500 mM D-Galactose, D-Mannose or D-Glucose showed no inhibitory effect on rHPL-LPS/bacteria interaction. Quantitative results were shown in Table 1. Since interaction between protein and monosaccharide is typically weak, a L-Rha conjugated BSA (Rha-BSA), was also measured. Rha-BSA was conjugated with ca.32 L-Rha per BSA molecule. It showed significantly stronger inhibitory effect on rHPL-LPS/bacteria interaction, such that 1 µM Rha-BSA could inhibit 73% to 80% of LPS/bacteria binding activities (Fig. 7 and Table 2). As for the negative control, BSA only, no inhibitory effect on rHPL-LPS/bacteria binding was observed (data not shown). These results indicated that rHPL reacted with Rha moiety and consequently reduced rHPL binding to LPSs or bacteria.

**Table 2:** Parameter of inhibitory effect on L-Rhamnose and L-Rhamnose-BSA conjugate to rHPL- LPS/bacteria interaction.

Inhibitor	LPS of *E. coli* O55:B5	LPS of *E. coli* O26:B6	LPS of *S.* typhimurium	LPS of *P. aeruginosa*	*P. aeruginosa* PAO1
**25 mM L-Rha**	74.5±7.2 *	87.2±11.9	80.6±0.74 *	83.6±2.4	72.8 ±6.3 **
**50 mM L-Rha**	73.5±4.1 **	70.8±8.7 **	66.3±6.6 ***	74.3±2.4 ***	48.4±8.5 ***
**100 mM L-Rha**	63.1±4.4 ***	54.1±5.7 ***	59.1±1.7 ***	69.0±7.3 ***	37.0±8.5 ***
**200 mM L-Rha**	53.2±7.4 ***	41.6±8.8 ***	44.5±3.4 ***	57.2±5.4 ***	30.4±4.5 ***
**500 mM L-Rha**	40.2±7.2 ***	21.2±4.8 ***	26.5±12.9 ***	44.7±7.8 ***	20.4±0.62 ***
**1 µM Rha-BSA**	28.6±0.5 ***	16.3±2.2 ***	33.3±4.5 ***	36.9±6.9 ***	18.3±0.47 ***

The values are the mean ± SD (%) from triplicate experiments.

*P<0.05, **P<0.01, and ***P<0.001 *versus* the rHPL only group (positive control).

Previous study indicated that TL-3 shows an 80% sequence identity with nTPL2 [12], and its hemagglutinating activity could be inhibited by blood group A-pentasaccharide through its GalNAcα1-3Gal structure [12]. However, this pentasaccharide showed no inhibitory effect on our rHPL-LPS/bacteria interaction (Fig. 7 and Table 1). Moreover, several glycans with similar structures to blood group A-pentasaccharide on CFG glycan array, such as glycan no. 83 and no. 418, also showed no interaction with our rHPL (data not shown). One of the reasons might arise from lacking of GalNAcα1-3Gal binding activity, a key feature of Japanese horseshoe crab TL-3, in our rHPL as indicated in CFG glycan array data.

### Antibacterial activity of rHPL

**Figure 8:**
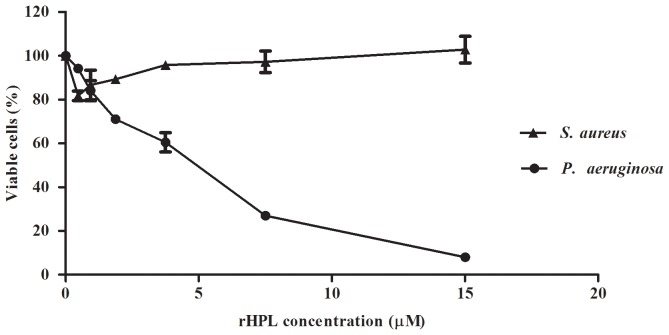
Antibacterial activity of rHPL. Samples of *P. aeruginosa* and *S. aureus* **were** mixed with rHPL at a final concentration of 0 µM (buffer only), 0.47 µM, 0.94 µM, 1.88 µM, 3.75 µM, 7.5 µM, and 15 µM and incubated at 37°C for 4 h. The antibacterial activity of rHPL was analyzed by plating serial dilutions of incubation mixture, and the CFUs were counted the following day. Control plate (0 µM) was defined as 100%-viable cells. Cell mortality was calculated as the decrease in the colony number compared with the control plate. The values are the mean ± SD from triplicate experiments.

Yeast expressed TPL2 showed antibacterial activity against *E. coli* Bos-12 [10]. To investigate the antibacterial activity of rHPL, *P. aeruginosa* PAO1 was examined. *S. aureus*, which was not recognized by rHPL (Fig. 3B), was also tested. A 25-µl sample of 1×10^6^ cells/ml bacteria was mixed with 25 µl of buffer without rHPL or with rHPL at a final concentration of 0 µM, 0.47 µM, 0.94 µM, 1.88 µM, 3.75 µM, 7.5 µM, and 15 µM, and incubated at 37°C for 4 h. The antibacterial activity of rHPL was analyzed by plating serial dilutions of the incubation mixture and counting the resulting CFUs the next day. rHPL inhibited *P. aeruginosa* PAO1 growth in a concentration-dependent manner, and the half-maximal inhibitory concentration was 4.3 µM (Fig. 8). No inhibitory effect was shown when *S. aureus* was treated with rHPL.

## Discussion

In this study, we generated rHPL, a recombinant form of TPL2 that is both soluble and functional. Purified rHPL not only retained the LPS- and bacterial-binding activities of TPL2 but also demonstrated binding activities to clinically isolated pathogens. Comparisons of the chemical structures of reported PAMPs on rHPL-interacting pathogens indicated that Rha was present on all the recognized samples. Additionally, rHPL could directly interact with Rha at the molecular level as demonstrated by a glycan array and MR assay, suggesting that the Rha moiety is a preferred ligand of rHPL.

Rha is a deoxy hexose found widely in bacteria and plants. It is a common component of the cell wall and capsule of many pathogenic bacteria, including Gram-negative *P. aeruginosa* [31], *S.* typhimurium [32], and *Vibrio cholerae* [33], [34], as well as *M. tuberculosis*, which is not classified as either Gram-positive or Gram-negative because it lacks the chemical characteristics of either [35]. Spores of *Bacillus anthracis* are also composed of Rha, which is required for pathogen interactions with macrophages [36]. In marine creatures there is a Rhamnose-binding lectin (RBL) family that specifically binds Rha and sugars possessing hydroxyls in the same configuration, i.e., axial-OH, equatorial-OH, and equatorial-OH for the carbons (C) C-2, C-3, and C-4, respectively, or for C-4, C-3, and C-2, respectively. RBLs are mainly distributed in the eggs and ovary cells of fish and invertebrates [37]–[39]. RBLs have been discovered in over 25 species of fish [37], [39]–[45], sea urchin [46], penguin wing oyster (*Pteria penguin*) [38], and ascidian (*Botryllus schlosseri*) [47]. Most RBLs possess two or three characteristic tandem-repeat CRDs (RBL-CRDs) consisting of ∼95 amino acid residues [48], [49]. Two characteristic peptide motifs, -(AN)YGR(TD)- (YGR-motif) and -DPCXGT(Y)KY(L)- (DPC-motif), which are located at the *N*- and *C*-terminal region of each domain, respectively, are conserved in almost all RBL-CRDs [49]. Sequence alignments between rHPL and the RBLs showed only limited similarities, and rHPL possessed neither the YGR- nor the DPC-motif (data not shown). Furthermore, the glycan array screening and the MR assay showed that rHPL could not interact with D-galactose, implying that rHPL might perform biological functions through its recognition of Rha-containing microbes that are similar to those of RBLs in marine organisms, albeit through different mechanisms.

In addition to bacterial recognition, rHPL specifically inhibited the growth of *P. aeruginosa*, an opportunistic nosocomial pathogen involved in a wide range of infections, which has high rates of antimicrobial resistance in immune-compromised individuals [50]. Rha is a common constituent found in the outer core of LPS of *P. aeruginosa* [51]–[53]. Rhamnolipids, virulence determinants secreted by *P. aeruginosa*, also contain Rha [54]. The Rha-binding activity of rHPL may contribute to the growth inhibitory effect on *P. aeruginosa.* Recently, drugs targeting cell wall synthesis, and thus Rha synthesis, in *M. tuberculosis* have been studied as possible clinical agents to treat tuberculosis [35]. Our engineered rHPL could be further developed as an antibacterial agent for pathogenic bacteria that have the Rha moiety on the cell surface, such as *M. tuberculosis*.

Most antibiotics, except polypeptide antibiotics, act by binding to enzymes involved in the biosynthesis of the cell wall and nucleic acids, or in protein synthesis. Antibiotics that bind to enzymes are highly specific, resulting in almost no serious side effects, but pathogens typically gain resistance to antibiotics by accumulating mutations [55]. In fact, abundant studies highlight the link between multidrug resistance and increased morbidity and mortality, increased lengths of hospitalization and higher hospital costs [56]. The mechanism of polypeptide antibiotics relies on binding to, and interfering with, cell wall synthesis (glycopeptides) or in altering bacterial outer membrane permeability by binding to the lipid A layer of LPS. The advantage of polypeptide antibiotics is that the major binding targets are metabolites of the pathogens [57], which means that pathogens cannot alter their membrane structures to avoid attacks by these polypeptide antibiotics.

LPS from Gram-negative bacteria also may cause sepsis or endotoxemia, allowing endotoxin to move into the patient's bloodstream. Measurement and treatment of endotoxemia are important for sepsis diagnosis and therapy. Each year, sepsis results in almost 250,000 deaths in the U.S. and costs the healthcare system more than $17 billion [58]. Because Rha is a common component of the cell wall and capsule of many pathogenic bacteria, Rha recognition may provide an alternative way for pathogen detection and inhibition in the future.

## Supporting Information

S1 Table Binding parameters of rHPL to LPSs.
(DOCX)

S2 Table Binding parameters of rHPL to laboratory-derived bacteria.
(DOCX)

S3 Table Binding parameters of rHPL to clinically isolated Gram-negative bacteria.
(DOCX)

S4 Table Binding parameters of rHPL to clinically isolated Gram-positive bacteria.
(DOCX)

S5 Table Magnetic reduction parameters of rHPL-conjugated MNPs titrated with analytes.
(DOCX)
