# Supplementary material for: A Recombinant Horseshoe Crab Plasma Lectin Recognizes Specific Pathogen-Associated Molecular Patterns of Bacteria through Rhamnose
Source: PLoS One. 2014 Dec 26;9(12):e115296. doi: 10.1371/journal.pone.0115296 (PMC4277298; doi:10.1371/journal.pone.0115296)
Supplement: S1 Table — Binding parameters of rHPL to LPSs. (DOCX) [file pone.0115296.s001.docx]

**Table S1**

| **Binding entity** | **OD_450_** | |
| --- | --- | --- |
|  | **Blank** | **rHPL** |
| *E. coli* O55:B5 | 0.081 ± 0.006 | 1.635 ± 0.259 *** |
| *E. coli* O26:B6 | 0.095 ± 0.012 | 2.091 ± 0.216 *** |
| *S.* typhimurium | 0.090 ± 0.019 | 2.246 ± 0.184 *** |
| *P. aeruginosa* | 0.118 ± 0.004 | 2.052 ± 0.427 *** |
