# Supplementary material for: A Recombinant Horseshoe Crab Plasma Lectin Recognizes Specific Pathogen-Associated Molecular Patterns of Bacteria through Rhamnose
Source: PLoS One. 2014 Dec 26;9(12):e115296. doi: 10.1371/journal.pone.0115296 (PMC4277298; doi:10.1371/journal.pone.0115296)
Supplement: S2 Table — Binding parameters of rHPL to laboratory-derived bacteria. (DOCX) [file pone.0115296.s002.docx]

**Table S2**

| **Binding entity** | | **OD_450_** | |
| --- | --- | --- | --- |
|  |  | **Blank** | **rHPL** |
| **Gram negative bacteria** | ***P. aeruginosa*** | 0.054 ± 0.002 | 1.502 ± 0.196 *** |
|  | ***S. flexneri*** | 0.058 ± 0.014 | 0.109 ± 0.171 |
|  | ***P. mirabilis*** | 0.069 ± 0.019 | 0.017 ± 0.219 |
|  | ***E. aerogenes*** | 0.040 ± 0.004 | 0.019 ± 0.065 |
|  | ***K. pneumoniae*** | 0.094 ± 0.083 | 0.073 ± 0.223 |
|  | ***S. marcescens*** | 0.137 ± 0.057 | 0.446 ± 0.109 |
|  | ***E. coli* TOP10 F'** | 0.063 ± 0.020 | 0.050 ± 0.095 |
| **Gram positive bacteria** | ***L. monocytogenes*** | 0.045 ± 0.003 | 1.246 ± 0.177 *** |
|  | ***S. aureus*** | 0.128 ± 0.072 | 0.375 ± 0.086 |
