# Supplementary material for: A Recombinant Horseshoe Crab Plasma Lectin Recognizes Specific Pathogen-Associated Molecular Patterns of Bacteria through Rhamnose
Source: PLoS One. 2014 Dec 26;9(12):e115296. doi: 10.1371/journal.pone.0115296 (PMC4277298; doi:10.1371/journal.pone.0115296)
Supplement: S3 Table — Binding parameters of rHPL to clinically isolated Gram-negative bacteria. (DOCX) [file pone.0115296.s003.docx]

**Table S3**

| **Binding entity** | | **Relative binding (%)** | **Binding entity** | | **Relative binding (%)** |
| --- | --- | --- | --- | --- | --- |
| **Negative control** | | 2.872 ± 0.363 | ***A. baumannii*** | #1 | 14.538 ± 0.694 |
| **Positive control** | | 100 *** |  | #2 | 9.077 ± 1.886 |
| ***S.* typhimurium** | Group B #1 | 10.627 ± 4.027 |  | #3 | 16.462 ± 2.542 |
|  | Group B #2 | 4.474 ± 0.334 |  | #4 | 9.744 ± 0.627 |
|  | Group B #3 | 4.908 ± 1.043 |  | #5 | 16.359 ± 0.887 |
|  | Group B #4 | 7.361 ± 0.946 |  | #6 | 13.436 ± 3.497 |
|  | Group B #5 | 6.616 ± 3.416 |  | #7 | 14.333 ± 4.637 |
| ***S.* cholerasuis** | Group C #1 | 20.166 ± 19.800 |  | #8 | 10.436 ± 2.908 |
|  | Group C #2 | 3.697 ± 1.055 |  | #9 | 4.154 ± 0.892 |
|  | Group C #3 | 4.034 ± 1.640 |  | #10 | 12.282 ± 13.310 |
|  | Group C #4 | 22.898 ± 22.917 |  | #11 | 3.385 ± 18.686 |
|  | Group C #5 | 3.582 ± 0.668 | ***P. aeruginosa*** | #S1 | 142.323 ± 5.227 *** |
| ***S.* enteritidis** | Group D #1 | 9.982 ± 0.466 |  | #S2 | 130.593 ± 4.870 *** |
|  | Group D #2 | 6.804 ± 0.308 |  | #S3 | 149.510 ± 20.640 *** |
|  | Group D #3 | 4.982 ± 0.290 |  | #S4 | 143.178 ± 7.985 *** |
|  | Group D #4 | 5.593 ± 0.668 |  | #S5 | 125.591 ± 6.852 *** |
|  | Group D #5 | 3.279 ± 1.363 |  | #R1 | 8.359 ± 3.592 |
| ***K. oxytoca*** | #1 | 21.125 ± 8.180 |  | #R2 | 112.308 ± 4.165 *** |
|  | #2 | 23.985 ± 5.256 |  | #R3 | 53.103 ± 6.441 *** |
|  | #3 | 133.179 ± 9.054 *** |  | #R4 | 84.205 ± 15.416 *** |
|  | #4 | 130.709 ± 9.790 *** |  | #R5 | 74.205 ± 2.879 *** |
