# Supplementary material for: A Recombinant Horseshoe Crab Plasma Lectin Recognizes Specific Pathogen-Associated Molecular Patterns of Bacteria through Rhamnose
Source: PLoS One. 2014 Dec 26;9(12):e115296. doi: 10.1371/journal.pone.0115296 (PMC4277298; doi:10.1371/journal.pone.0115296)
Supplement: S4 Table — Binding parameters of rHPL to clinically isolated Gram-positive bacteria. (DOCX) [file pone.0115296.s004.docx]

**Table S4**

| **Binding entity** | | **Relative binding (%)** |
| --- | --- | --- |
| **Negative control** | | 2.872 ± 0.363 |
| **Positive control** | | 100 *** |
| ***S. aureus*** | #1 | 5.715 ± 1.022 |
|  | #2 | 3.598 ± 2.263 |
|  | #3 | 5.525 ± 4.231 |
|  | #4 | 8.356 ± 2.697 |
|  | #5 | 8.019 ± 1.783 |
| ***S. pneumoniae*** | Serotype 3 #1 | 7.293 ± 1.073 |
|  | Serotype 3 #2 | 11.424 ± 1.567 |
|  | Serotype 3 #3 | 10.877 ± 2.558 |
|  | Serotype 3 #4 | 16.962 ± 10.530 |
|  | Serotype 3 #5 | 16.023 ± 9.974 |
|  | Serotype 14 #1 | 9.635 ± 0.467 |
|  | Serotype 14 #2 | 10.089 ± 2.276 |
|  | Serotype 19A #1 | 29.329 ± 14.308 * |
|  | Serotype 19A #2 | 25.818 ± 12.122 |
|  | Serotype 19A #3 | 27.232 ± 15.192 |
|  | Serotype 19B #1 | 119.682 ± 3.064 *** |
|  | Serotype 19F #1 | 121.353 ± 1.955 *** |
|  | Serotype 19F #2 | 135.401 ± 12.721 *** |
|  | Serotype 19F #3 | 110.084 ± 5.806 *** |
|  | Serotype 19F #4 | 116.278 ± 12.575 *** |
|  | Serogroup 23F #1 | 31.056 ± 15.820 * |
|  | Serogroup 23F #2 | 15.581 ± 4.371 |
