# Supplementary material for: A Recombinant Horseshoe Crab Plasma Lectin Recognizes Specific Pathogen-Associated Molecular Patterns of Bacteria through Rhamnose
Source: PLoS One. 2014 Dec 26;9(12):e115296. doi: 10.1371/journal.pone.0115296 (PMC4277298; doi:10.1371/journal.pone.0115296)
Supplement: S5 Table — Magnetic reduction parameters of rHPL-conjugated MNPs titrated with analytes. (DOCX) [file pone.0115296.s005.docx]

**Table S5**

| **Analyte concentration (ng/ml)** | **Magnetic reduction rate (%)** | | |
| --- | --- | --- | --- |
|  | **D-Galactose** | **L-Rhamnose** | **LPS of *P. aeruginosa*** |
| **0.01** | 2.62 ± 0.02 | 3.73 ± 0.31 | 3.10 ± 0.35 |
| **0.1** | 1.92 ± 0.23 | 4.13 ± 0.32 | 3.20 ± 0.30 |
| **1** | 2.14 ± 0.18 | 5.4 ± 0.40 | 4.32 ± 0.42 |
| **10** | 2.61 ± 0.02 | 8.00 ± 0.42 | 8.17 ± 0.42 |
| **100** | 3.27 ± 0.89 | 10.85 ± 0.78 | 10.08 ± 0.59 |
| **1000** | 2.14 ± 0.18 | 11.43 ± 0.93 | 10.20 ± 0.58 |
